# Correction: Japanese-Language AI Agent System for Human Papillomavirus Vaccine Infoveillance and Public Communication: Development and Feasibility Evaluation

**DOI:** 10.2196/109982

**Published:** 2026-10-28

**Authors:** Junyu Liu, Siwen Yang, Dexiu Ma, Qian Niu, Zequn Zhang, Momoko Nagai-Tanima, Tomoki Aoyama

**Affiliations:** 1 Kyoto University, Graduate School of Medicine, Yoshida-honmachi, Sakyo-ku, Kyoto, 606-8501, Japan, 81 075-753-7531; 2 David R. Cheriton School of Computer Science, University of Waterloo, Waterloo, ON, Canada; 3 Department of Computer Science, Whitacre College of Engineering, Texas Tech University, Lubbock, TX, United States; 4 Graduate School of Engineering, The University of Tokyo, Bunkyo-ku, Tokyo, Japan; 5 Department of EEIS, University of Science and Technology of China, Hefei, Anhui, China

 In “Japanese-Language AI Agent System for Human Papillomavirus Vaccine Infoveillance and Public Communication: Development and Feasibility Evaluation” [1], we identified several items requiring correction. They are set out below in the order in which they appear in the article, providing the original published text and the corrected text. None of the corrections change any finding or conclusion of the article.

First, in the author byline, the degree designations of three authors were incorrect. The byline originally read:

*Junyu Liu, PhD; Siwen Yang, BEng; Dexiu Ma, PhD; Qian Niu, PhD; Zequn Zhang, PhD; Momoko Nagai-Tanima, PhD; Tomoki Aoyama, MD, PhD*

The corrected byline now reads:

*Junyu Liu, ME; Siwen Yang, BEng; Dexiu Ma, MS; Qian Niu, PhD; Zequn Zhang, MS; Momoko Nagai-Tanima, PhD; Tomoki Aoyama, MD, PhD*

Second, in the section Report Generation Evaluation Methodology in the Methods section, the following text was corrected:

*Both protocols use LLM-based judges with standardized scoring rubrics, generating scores on a scale from 0 to 5 (Multimedia Appendices 4 and 5) for each dimension.*

The text now reads:

*Both protocols were applied by 3 domain experts using the standardized scoring rubrics provided in Multimedia Appendices 4 and 5, assigning scores on a scale from 0 to 5 for each dimension. Each expert scored every report section independently.*

This aligns the paragraph with the remainder of the same section (“Three volunteers read and scored each report independently”) and with the Results section (“Three evaluators independently scored each report section”), which are correct. The values reported in Table 5 and Multimedia Appendix 10 (included here as Multimedia Appendix 1) are the scores of these 3 raters. An automated large language model–based checker was also implemented and executed, but its output was not used for the values reported in this article.

This correction does not affect the corresponding sentence in the section Chatbot Evaluation Methodology, where the chatbot evaluation was indeed performed by large language model–based judges, as stated.

Third, in the Social Media Analysis subsection of the Methods, the stance classifier assigns one of three labels; no “unclear” label is produced. The sentence, which read:

Stance classification categorizes each post as supportive, opposed, neutral, or unclear regarding HPV vaccination using batch LLM inference with a temporal context, thereby aggregating daily counts to track sentiment evolution.

has been corrected to:

Stance classification categorizes each post as supportive, opposed, or neutral regarding HPV vaccination using batch LLM inference with a temporal context, thereby aggregating daily counts to track sentiment evolution.

The daily counts reported in the article were aggregated over the three labels the classifier assigns, so no reported value changes.

In the same section, misinformation detection flags a post and records a short statement of the claim; it does not assign the post to a type. The sentence, which read:

Misinformation detection uses LLM-based analysis to identify posts containing claims that contradict the established scientific consensus, categorizing detected content by type (safety concerns, efficacy doubts, and conspiracy theories) for institutional awareness.

has been corrected to:

Misinformation detection uses LLM-based analysis to identify posts containing claims that contradict the established scientific consensus, recording for each flagged post a short statement of the claim for institutional awareness.

No counts by type are reported in the article, so no reported value changes.

Fourth, the row for chat conversations in Table 1 described the set of conversations used for the chatbot evaluation, but the three values in the row were derived from three different populations. The row was published as:

**Table:** 

Collection	Documents (n=139,803), n (%)	Unique source	Primary language
Chat conversations	139 (0.10)	31 sessions	Japanese (59.3%)

The table row has been corrected to the following:

**Table:** 

Collection	Documents (n=139,803), n (%)	Unique source	Primary language
Chat conversations	139 (0.10)	37 sessions	English (98.6%); Japanese (1.4%)

The following footnote has been added to Table 1:

*The chat row describes the conversation set used for the chatbot evaluation: 139 turns, each stored as one document, from 37 conversations. Language proportions were computed over the 139 user messages in this set. Primary language for the Papers, Official documents and Social media rows was assigned on the basis of the source of each collection and was not derived from automated language detection.*

The other three rows of Table 1, the column percentages, and the total of 139,803 documents are unchanged. Specifically, the number of sessions has been corrected from 31 to 37. The 139 documents are the 139 single-turn question-answer exchanges reported in Table 2, which originate from 37 conversations (Multimedia Appendix 6). The figure of 31 is the number of conversations used for the multiturn evaluation reported in Table 3 (Multimedia Appendix 7), which comprise 133 turns.

The language proportions have been corrected. Computed over the 139 user messages in this set, 137 (98.6%) are in English and 2 (1.4%) are in Japanese. The previously reported value of “Japanese (59.3%)” was derived from a different population and does not describe the 139 documents referred to in this row.

Fifth, the multiturn evaluation block of Table 4 was published as:

**Table:** 

Check type and dimension	Rater 1	Rater 2	Rater 3	MAD^a^	Spearman ρ (95% CI)	ICC^b^(3,1) (95% CI)
Multiturn evaluation (n=11 conversations)						
Context memory	0.13	0.13	0.13	0.13	—	1.000 (1.000 to 1.000)
Topic centering	0	0.13	0	0.04	—	0.000 (−0.307 to 0.545)
Identity	0	0.13	0.55	0.21	—	0.174 (−0.208 to 0.684)

Table 4 has been corrected as follows:

**Table:** 

Check type and dimension	Rater 1	Rater 2	Rater 3	MAD^a^	Spearman ρ (95% CI)	ICC^b^(3,1) (95% CI)
Multiturn evaluation (n=8 conversations)						
Context memory	0.13	0.13	0.13	0.13	—	—^e^
Topic centering	0	0.13	0	0.04	—	0.000 (−0.307 to 0.545)
Identity	0	0.13	0.50	0.21	—	0.174 (−0.208 to 0.684)

The new footnote e reads:

^*e*^ *The 3 experts’ scores were all the same, and the ICC could not be calculated.*

All statistics in the multiturn block of Table 4 were computed over these 8 conversations; the header overstated the number. For the Context memory row, all 3 experts assigned a score of 5 to every conversation, so the between-item variance is zero and the intraclass correlation coefficient is undefined; the em dash follows the convention already used in the Spearman ρ column of the same table.

The single-turn evaluation block of Table 4 is unchanged.

Sixth, in the Results section, the following text was revised:

*n=20 for single-turn evaluation and n=11 for multiturn evaluation*

The text has been corrected to:

*n=20 question-answer pairs for single-turn evaluation and n=8 conversations for multiturn evaluation*

The corrected text also names the unit of each evaluation set.

Seventh, in the Results section, the following sentence was corrected:

*Context memory showed perfect interrater reliability (ICC=1.000) as all 3 experts assigned identical scores, whereas topic centering (ICC=0.000) and identity (ICC=0.174) showed that the small deviations among raters did not follow consistent patterns—a consequence of near-ceiling score distributions.*

The text now reads:

*All 3 experts assigned identical scores to every conversation for context memory, whereas topic centering (ICC=0.000) and identity (ICC=0.174) showed that the small deviations among raters did not follow consistent patterns—a consequence of near-ceiling score distributions.*

This aligns the sentence with the correction to the ICC(3,1) column in item 5.

Eighth, in the section Report Generation in the Results, the following text was corrected:

*Citation correctness was the most variable dimension (median 4.00, IQR 4.00‐5.00; mean 4.21, 95% CI 3.96‐4.46), with scores spanning the range of 4 to 5*

The text now reads:

*Citation correctness was the most variable dimension (median 4.00, IQR 4.00‐5.00; mean 4.21, 95% CI 3.96‐4.46), with scores spanning the range of 3 to 5*

Ninth, in Table 5, the rows for Helpfulness and Citation correctness originally read:

**Table:** 

Dimension	Score (0‐5), mean (SD; 95% CI)	Score (0‐5), median (IQR)	Score of 5, %	Score of ≥4, %	Score of ≤3, %
Helpfulness	4.12 (3.98‐4.27)	4.00 (4.00‐4.00)	19	94	0
Citation correctness	4.21 (3.96‐4.46)	4.00 (4.00‐5.00)	29	92	0

The rows have been corrected to the following:

**Table:** 

Dimension	Score (0‐5), mean (SD; 95% CI)	Score (0‐5), median (IQR)	Score of 5, %	Score of ≥4, %	Score of ≤3, %
Helpfulness	4.12 (3.98‐4.27)	4.00 (4.00‐4.00)	19	94	6
Citation correctness	4.21 (3.96‐4.46)	4.00 (4.00‐5.00)	29	92	8

The published values in the “Score of ≤3, %” column were inconsistent with the “Score of ≥4, %” column in the same rows. The corrected values correspond to 3 of the 48 helpfulness scores and 2 of the 24 citation correctness scores being 3 (3/48, 6%; 2/24, 8%). The other three rows of Table 5 are unchanged.

Tenth, in the section Interpretation in the Discussion, the following sentence has been deleted:

*The higher agreement in multiturn evaluation, where full dialogue context is available (context memory ICC=1.000), supports this interpretation.*

Eleventh, in the section Limitations in the Discussion, the following text was revised:

*The evaluation dataset (31 conversations and 139 exchanges) may not capture all real-world interaction patterns*

This has been corrected to the following:

*The evaluation datasets (139 single-turn exchanges and 31 multiturn conversations) may not capture all real-world interaction patterns*

The two figures in the original text described different evaluation sets: the 139 exchanges are the single-turn evaluation items (Multimedia Appendix 6), whereas the 31 conversations are the multiturn evaluation set (Multimedia Appendix 7), which comprises 133 turns. As printed, the two figures could be read as the conversation and turn counts of a single set.

Twelfth, in the Conclusions section of the Discussion, the following text was revised:

*The integrated architecture may enable bidirectional information flow—providing verified information with transparent source attribution to users while generating analytical reports for institutional stakeholders—creating feedback loops between public concerns and communication strategies.*

The text has been corrected to:

*The integrated architecture may enable bidirectional information flow—providing verified information with transparent source attribution to users while generating analytical reports for institutional stakeholders—creating links between public concerns and communication strategies.*

Only the term “feedback loops” has been changed. The term was used to refer to the circulation of information: conversations recorded with user consent become available to the report generation component so that public concerns can reach institutional readers. However, “feedback” also carries its control-theoretic sense, in which an output returns to the input and automatically adjusts the behavior of the system. The system implements no such loop: no path from the generated reports back to the conversational interface exists. Because the two senses can be confused, the term was not an appropriate choice. “Links” states the relationship without implying closure or automatic adjustment. No reported result is affected.

Thirteenth, the authors have replaced Multimedia Appendix 10 (included here as Multimedia Appendix 1) with a new file. Three corrections have been made to it.

In the table of reference evaluation results by section, the column headers “Completeness” and “Correctness” have been corrected to “Reference validity” and “Citation correctness,” respectively. The headers as published were those of the main text evaluation table above it; the columns contain reference validity and citation correctness scores.

In the same table, the value for July 2020 / Paper / Citation correctness has been corrected from “4.00±0.00” to “4.00 ± 1.00.” The three expert scores for this cell were 3, 4, and 5. This is the only cell of that table whose value did not reproduce; the other 15 are unchanged.

In the same table, the “Overall” values for Citation correctness have been corrected from “4.33±0.50” to “4.33±0.49” (News) and from “4.08±0.66” to “4.08±0.67” (Paper). The corresponding means are correct as published.

The SDs in Multimedia Appendix 10 (included here as Multimedia Appendix 1) are sample SDs, consistent with the formulas in Multimedia Appendix 9 from which the table was produced. The following footnote has been added to both tables of the appendix file:

*Values are mean±sample standard deviation across the 3 evaluators, computed from the scores in Multimedia Appendix 9. The standard deviations reported in the Abstract are population standard deviations.*

None of these corrections affects the values reported in Table 5 or any finding of the study.

Lastly, the Acknowledgments originally read:

*The authors used the generative artificial intelligence tool Claude [33] by Anthropic to refine the writing and structure of the manuscript, which was further reviewed and revised by the study group. The original Claude transcripts are available in Multimedia Appendix 11.*

This has been corrected to the following:

*The authors used the generative artificial intelligence (AI) tool Claude [33] by Anthropic to generate code, to assist with the analysis, to search the literature for references supporting statements in the manuscript, to retrieve reported figures from cited sources, and to refine the writing and structure of the manuscript. All AI-generated output was reviewed and revised by the study group. A record of part of these interactions is provided in Multimedia Appendix 11. The remaining interactions were not retained, so a complete transcript is not available.*

Two aspects of the original statement required correction. First, it described the use of the tool as limited to refining the writing and structure of the manuscript. The tool was in fact also used to generate code, to assist with the analysis, to search for literature supporting statements in the manuscript, and to retrieve figures from cited sources; the corrected text states this. Second, “the original Claude transcripts are available” could be read as indicating that the appendix contains the complete record. Multimedia Appendix 11 consists of 18 screenshots of part of these interactions; the remainder was not saved and cannot be supplied.

The reported values do not depend on the correctness of any generated code: 280 reported values—those in Tables 1-5 and Multimedia Appendix 10 (included here as Multimedia Appendix 1), together with the associated statistics and bootstrap CIs reported in the text—have been independently recomputed from the saved evaluation data and the underlying document collections. Of these, 271 reproduce the values as published; the 9 that do not are precisely the numerical corrections in items 4, 5, 9, and 13 above. The CIs reproduce exactly under the random seed fixed in the analysis code.

All coauthors have reviewed and approved these corrections and the replacement appendix file. We can provide their written agreement, a cell-level list of the changed appendix values, and the verification scripts and their output on request.

The correction will appear in the online version of the paper on the JMIR Publications website together with the publication of this correction notice. Because this was made after submission to PubMed, PubMed Central, and other full-text repositories, the corrected article has also been resubmitted to those repositories.

## Supplementary material

10.2196/109982 Multimedia Appendix 1 Correction of Multimedia Appendix 10: Section-level evaluation results for the generated reports.
